# SARS‐CoV‐2 and immunosuppressors at cellular level: Some good news

**DOI:** 10.1002/ueg2.12427

**Published:** 2023-06-20

**Authors:** Candida Abreu

**Affiliations:** ^1^ Department of Infectious Diseases São João Hospital Center Porto Portugal; ^2^ Department of Medicine Faculty of Medicine University of Porto Porto Portugal; ^3^ Instituto de Inovação e Investigação em Saúde (I3S) Porto Portugal

**Keywords:** immunology, immunomodulators, immunosuppressors, inflammatory bowel disease, molnupiravir, nirmatrelvir/ritonavir, SARS‐CoV‐2 infection

In their recent manuscript, Wang Y and colleagues focused on the effects of immunomodulators and immunosuppressors on pan‐coronavirus infections resorting to experimental models, namely lung cell lines and human airway organoids (hAOs).^1^ This “back to basics” approach explored the mechanisms of pathogenicity of coronaviruses under immunosuppressors and the response to antivirals, avoiding the complexity of the studies in real patients and providing evidence for new therapies. Lung cells and hAOs were inoculated with several SARS‐CoV‐2 variants and common coronaviruses and treated with different concentrations of 15 drugs, including 13 common immunosuppressors (6‐mercaptopurine, azathioprine, methotrexate, tofacitinib, filgotinib, budesonide, dexamethasone, prednisolone, everolimus, cyclosporin A, tacrolimus, mycophenolic acid, 6‐thioguanine, rapamycin), 5‐aminosalicylic acid (5‐ASA) and sulfasalazine. The results revealed that dexamethasone and 5‐ASA promoted moderate coronavirus replication.

From a clinical perspective, patients with COVID‐19 and immune‐mediated inflammatory diseases who were treated with corticosteroids have been shown to have a higher risk of hospitalization and mortality.^2^, ^3^, ^4^ However, corticosteroid therapy has also been shown to have positive outcomes in inflammation‐related lung diseases^5^ and in COVID‐19 by reducing inflammation‐coagulation‐fibroproliferation, with reduction of morbidity and mortality,^6^ which may seem paradoxical. These ambiguous effects might be partly due to the timing and dosage of corticosteroid use in the timeline of SARS‐CoV‐2 infection.

Brenner et al.^7^ found that in IBD patients with COVID‐19, adjusted for age, comorbidities, inflammatory bowel disease (IBD) disease characteristics, and corticosteroids, the use of 5‐ASA was positively associated with the outcome “hospitalization or death” (adjusted odds ratio 3.1; 95% CI 1.3–7.7). Still, SECURE‐IBD data, which included 6144 reports received between 13 March 2020 and 21 May 2021, indicated that 5‐ASA and sulfasalazine were not associated with worse outcomes.^8^ Research has evidenced that these compounds exert their anti‐inflammatory effects by acting on peroxisome proliferator‐activated receptor gamma (PPAR‐γ) receptors. However, PPAR‐γ also upregulates the expression of angiotensin‐converting enzyme 2 (ACE2),^9^, ^10^ which is the binding receptor for SARS‐CoV‐2. Therefore, the potential for 5‐ASA to worsen clinical outcomes in patients with COVID‐19 is still a matter of debate.

The study also revealed that mycophenolic acid (MPA), 6‐thioguanine (6‐TG), tofacitinib, and filgotinib inhibited viral replication of all tested coronaviruses in a dose‐dependent manner.

Along with antiviral agents, immunosuppressive agents have been used to prevent and treat COVID‐19‐associated cytokine storms, which are one of the main causes of mortality during SARS‐CoV‐2 infection. In COVID‐19 hospitalized patients receiving supplemental oxygen therapy or mechanical ventilation, treatment with glucocorticoids and other anti‐inflammatory agents, in particular JAK inhibitors such as tofacitinib and baracitinib, was found to provide benefits concerning disease management.^11^, ^12^

Upon COVID‐19 diagnosis, discontinuing drugs essential for treating severely immunosuppressed patients (like transplant recipients or hematological patients) may be worrisome. In this setting, the findings of Wang Y and colleagues regarding the effect of mycophenolic acid and 6‐TG on the inhibition of coronavirus replication are relevant. A recent study with COVID‐19 patients found that the use of mycophenolate reduced mortality and duration of hospital stay compared with the standard of care.^13^ Due to its anti‐proliferative properties, mycophenolate is essential to reduce the risk of organ rejection in transplanted patients, and therefore, its discontinuation after COVID‐19 diagnosis may have serious consequences.

A key knowledge of the present study is the relationship of SARS‐CoV‐2 with the JAK‐STAT3 pathway. The virus has been shown to activate STAT3 in cell models and in kidney and lung tissues of COVID‐19 patients. As such, JAK inhibitors, mainly those that target the STAT3 pathway, may be particularly effective as COVID‐19 treatment due to their combined action on the virus and in cytokine storms.

Finally, the study showed a dose‐dependent additive or synergistic antiviral activity effect of the combination of MPA, 6‐TG, tofacitinib, and filgotinib with the antivirals nirmatrelvir/ritonavir and molnupiravir. This observation seems to indicate that antiviral COVID‐19 treatment in patients on these immunosuppressors is not only possible but also desirable.

The findings of Wang Y and colleagues may sound like good news for COVID‐19 patients under these immune suppressors. However, basic studies on SARS‐CoV‐2 infection in the context of immunosuppression are welcomed. In fact, basic and clinical investigations are warranted to deepen the knowledge on COVID‐19 infection and to further clarify clinical data and respective confounders. To finalize, at this point, it is clear that SARS‐CoV‐2 infection should no longer be approached as an opportunistic infection.

## CONFLICT OF INTEREST STATEMENT

The authors have no conflicts of interest to declare.

## FUNDING INFORMATION

The author received no funding for this research and authorship.

## Data Availability

Data sharing is not applicable to this article as no new data were created or analyzed in this study.
